# Fear of COVID‐19: A Comparative Study Among University Students in Peru

**DOI:** 10.1155/tswj/4379030

**Published:** 2026-07-02

**Authors:** Carmen Y. Baltazar-Meza, María Custodio, Patricia Roxana Zarela Quispe-Baltazar, Carmen Soledad Mejía-Murillo, Flor Del Rosario Díaz-Díaz, Ciro Lazo-Salcedo

**Affiliations:** ^1^ Academic Department of Education, National University of Central Peru, Huancayo, Peru; ^2^ Academic Department of Human Medicine, National University of Central Peru, Huancayo, Peru; ^3^ Mirones Bajo Community Mental Health Center, MINSA-DIRIS, Lima, Peru; ^4^ Collage of Education and Humanities, San Pedro University, Chimbote, Peru; ^5^ Academic Department of Philosophy and Art, National University of Trujillo, Trujillo, Peru, unitru.edu.pe; ^6^ Academic Department of Social Sciences and Humanities, Hermilio Valdizán National University, Huánuco, Peru

**Keywords:** COVID-19, fear of COVID-19, health crisis, mental health, psychological symptoms, university students

## Abstract

The COVID‐19 pandemic severely affected university students′ mental health. The objective of this study was to evaluate the level of fear associated with COVID‐19 in university students from the Coast, Sierra, and the Jungle regions of Peru between April and May 2020. The sample consisted of 915 university students from the three regions of Peru. The Fear of COVID‐19 Scale was used to assess fear and its dimensions (psychological and somatic symptoms). Data were analyzed using analysis of variance (ANOVA) and Tukey′s post hoc test to compare group means. Additionally, multiple linear regression analysis was used to identify significant predictors of fear. Results showed a moderate overall level of fear, with significant differences by sex and geographic region. Women reported significantly higher levels of fear than men (*p* < 0.001), suggesting a disproportionate psychological impact on this group. Furthermore, students from the Coast reported significantly higher levels of psychological symptoms of fear compared to those from the Sierra and Jungle regions (*p* < 0.001). Multiple linear regression analysis identified sex, region, and field of study as significant predictors of fear (*p* < 0.05). These findings confirm that fear of COVID‐19 had a prominent and differentiated psychological impact on the Peruvian university population. These results underscore the need to implement targeted intervention strategies and mental health programs that consider variations by sex and region in future health crisis contexts.

## 1. Introduction

The fear of COVID‐19 accentuated global inequalities and health vulnerabilities [1–3], increasing mortality and deteriorating global mental health by increasing depression and disrupting basic routines [4–7]. This impact was critical for university students, whose mental health was affected by emotional isolation [8]. Research in 36 countries associates fear of the virus with high rates of anxiety and depression [9]. Sudden transition to virtual learning, academic uncertainty, and lack of face‐to‐face interaction created an environment of severe psychological distress [10, 11]. Studies in China revealed symptoms of anxiety in more than half of the student body [12], whereas in England, academic stress increased depression rates by 50% [13]. Overall, the literature confirms a disproportionate psychological burden on students derived from the disruption of their traditional academic and social lives [14, 15].

Fear of COVID‐19 has been identified as a psychological phenomenon distinct from general anxiety or stress, which decisively influenced behavior and well‐being during the pandemic [16]. This intense concern for one′s own health and that of loved ones, manifested as avoidance behaviors, is directly linked to the appearance of somatic and psychological symptoms [17, 18]. Unlike acute fear, which can be adaptive to an immediate threat, prolonged exposure to a perceived danger (COVID‐19 pandemic) generates psychological, physiological and anatomical changes, increasing the risk of developing anxiety disorders, depression and posttraumatic stress [19].

The World Health Organization (WHO) has documented a deterioration in mental health, identifying university students as a particularly susceptible group. Global studies report a high prevalence of depression, anxiety, and stress, which increased significantly after the onset of the pandemic [20, 21]. These findings highlight student vulnerability and the need to understand coping mechanisms in the face of fear. Social disruptions and changes in interaction profoundly impacted their well‐being [22]. In Japan, virtual education was associated with increased anxiety [23].

In Peru, one of the countries most severely affected by the pandemic, the scarcity of local research during the first wave hindered the development of timely intervention strategies [24, 25]. Although previous studies in Peru have explored these issues, the majority were limited to specific populations, such as healthcare personnel or health science students [26]. Consequently, there is a critical need for research that encompasses a broader, more heterogeneous demographic. This study addresses this gap by analyzing a diverse sample of students from various academic fields across the country′s three distinct geographic regions: the Coast, the Sierra, and the Jungle. Therefore, the objective of this study was to evaluate the level of fear associated with COVID‐19 in university students from the Coast, Sierra, and the Jungle regions of Peru between April and May 2020.

## 2. Materials and Methods

### 2.1. Design and Selection of Participants

A descriptive, cross‐sectional, and comparative study was conducted among university students in Peru during the 2020‐I academic period, specifically between April and May 2020. Due to the restrictions imposed by the pandemic, data were collected through an online questionnaire, which was distributed by the researchers via social media (WhatsApp) and email. The participants were students from the faculties of education, health sciences, and engineering in the three regions of Peru (the Coast, the Sierra, and the Jungle).

### 2.2. Sample Size

The sample was selected using convenience sampling due to the restrictions imposed by the state of health emergency. This type of sampling was essential to collect data quickly and safely, given that it was necessary to capture the psychological impact at a critical moment in the pandemic. Despite being a nonprobability sample, the size of 915 participants is considerable, providing the study with sufficient statistical power. The participation of students from the Coast, the Sierra, and the Jungle regions contributed to a geographically relevant representation in the Peruvian context during that period.

### 2.3. Data Collection

Data collection took place during the first wave of the COVID‐19 pandemic in Peru, a period characterized by strict national lockdown measures. Given the restrictions on movement, an online survey was chosen as the only viable way to reach the university population. A snowball sampling strategy was used to distribute the questionnaire, which was hosted via Google Forms. The link was shared on social media and via email, allowing participants to share the questionnaire with their peers. This methodology facilitated the participation of 915 Peruvian university students from the Coast, Sierra, and the Jungle regions. Of the total sample, 78.0% (*n* = 714) were women, indicating that the sample is not balanced by gender. In addition to questions related to fear of COVID‐19, key sociodemographic and academic data were collected for analysis, including age, sex, academic year, and region of origin of the participants. The inclusion criteria were enrollment as a college student residing in the selected regions, providing informed consent, and completing the questionnaire in its entirety; therefore, all incomplete responses were excluded. Notably, no additional psychological variables, such as personality or coping strategies, were assessed.

### 2.4. Data Quality Control

Since data collection was conducted via an online survey during lockdown, rigorous measures were implemented to ensure quality. The survey platform was configured with validation rules requiring all mandatory questions to be answered, which guaranteed data integrity and eliminated missing values. To prevent multiple entries, IP (Internet Protocol) addresses were monitored to detect and eliminate duplicate responses. Finally, a manual inspection was carried out to identify and discard responses with inconsistent patterns or implausible survey completion times that indicated a lack of participant attention. These steps were essential to minimize selection bias and enhance the overall reliability of the findings.

### 2.5. Measurement Instrument

Fear of COVID‐19 Scale (FCV‐19S) was used to assess fear of COVID‐19. This was a unidimensional scale comprising seven items. It was selected because it was one of the first to be developed and was widely adopted globally during the first wave of the pandemic. Its selection was based on the robust psychometric properties demonstrated in various previous studies [27, 28]. Although the scale is unidimensional, this study considers two factors for analysis: a psychological symptoms factor (Items 1, 2, 4, and 5) and a somatic symptoms factor (Items 3, 6, and 7). Responses were recorded on a 5‐point Likert scale, where 1 represented “*strongly disagree*” and 5 represented “*strongly agree*.” The possible total score range was 7–35 points. The reliability of the scale was assessed using Cronbach′s alpha coefficient, showing excellent overall internal consistency (*α* = 0.887). The internal consistency of the subscale was also robust, with values of *α* = 0.848 for the psychological symptoms subscale and *α* = 0.845 for the somatic symptoms subscale.

### 2.6. Ethical Considerations

This study was conducted in accordance with the procedures regulated by the Code of Ethics for Researchers of the National University of Central Peru (Resolution No. 4600‐CU‐2018). All participants were informed of the purpose of the study, and their consent was obtained before administering the questionnaire. They were also informed that their consent could be revoked at any time. The confidentiality of the information collected was strictly maintained, and no personal data, such as names or other identifying information, was recorded. Participation in the study was completely voluntary, and participants were informed that they could revoke their consent at any time.

### 2.7. Statistical Analysis

Statistical analysis of the data was performed using IBM SPSS 29.0 software. Descriptive statistics were used to calculate frequencies and percentages in the characterization of the sample. The internal reliability of the measurement scale was assessed using Cronbach′s alpha coefficient. The Student′s *t*‐test was applied to compare fear of COVID‐19 between two groups (e.g., by sex). In the case of variables with three or more groups, a one‐way analysis of variance (ANOVA) was used. Subsequently, Tukey′s HSD test was performed as a post hoc analysis to identify which specific groups had statistically significant differences. The linear relationship between continuous variables was examined using Pearson′s correlation coefficient (*R*). The value of *p* < 0.05 was established as the significance threshold in all analyses. Subsequently, multiple linear regression analysis was performed to predict fear of COVID‐19 and identify the relative contribution of the various factors analyzed. This approach was selected for its ability to model complex relationships, allowing us to examine several variables (sex, age, region, etc.) that simultaneously influence the level of fear. This is essential, as fear is rarely caused by a single factor. This analysis helped isolate the unique effect of each variable, providing a more accurate understanding of its individual impact. It also allows us to identify the specific contribution of each predictive variable. This predictive ability is essential for designing more effective mental health interventions and policies.

## 3. Results

### 3.1. Sociodemographic Characteristics of Participants

A survey on fear of COVID‐19 was administered to university students from the three geographical regions of Peru (Coast, Sierra, and Jungle). Of the 915 participants, 78.00% were women, mainly students from the Sierra. In terms of the academic year completed by the students, there was a relatively wide distribution across the 5 years, with percentages ranging from 12.90% (*n* = 118) for the first year to 25.90% (*n* = 237) for the third year, with no marked concentration in a single year (Table 1). The age distribution revealed that the predominant group was between 18 and 25 years old.

**Table 1:** Characteristics of the sample of Peruvian university students (*n* = 915).

Variable	Group	Frequency	Percentage (%)
Sex	Female	714	78.00
	Male	201	22.00
Age	Under 18	18	2.00
	Between 18 and 25	755	82.50
	Between 26 and 35	121	13.20
	Over 35	21	2.30
Region	Sierra	658	71.90
	Coast	173	18.90
	Jungle	84	9.20
Area of study	Education and humanities	789	86.20
	Health sciences	41	4.50
	Engineering	85	9.30
Academic year	First	118	12.90
	Second	217	23.70
	Third	237	25.90
	Fourth	197	21.50
	Fifth	146	16.00

### 3.2. Levels of Fear of COVID‐19 and Their Variation According to the Characteristics of Peruvian University Students

Participants in this study reported a moderate level of fear of COVID‐19 (Table 2). Comparison of perceived fear between men and women revealed a significant difference (*t* = 3.223, *p* = 0.001). Females (mean = 20.37, SD = 6.04) perceived a significantly higher level of fear of COVID‐19 compared to males (mean = 18.80, SD = 6.33), confirming the trend observed in the overall study. The one‐way ANOVA also showed a significant difference in fear of COVID‐19 between groups belonging to different geographic regions (*F* = 3.949, *p* = 0.020). A post hoc analysis using Tukey′s range test identified that participants from the Coast experienced a significantly higher level of fear compared to the other participants. In contrast, no statistically significant differences in perceived fear were found between the different study areas or between the participants′ academic years.

**Table 2:** Comparison of fear of COVID‐19 between groups for each variable.

Variable	Group	Mean	SD	*F* or *t*	*p*
Sex	Female	20.37	6.04	3.223	0.001
	Male	18.80	6.33		
Age	Under 18	19.17	4.79	0.418	0.740
	Between 18 and 25	20.07	6.04		
	Between 26 and 35	19.73	6.81		
	Over 35	21.05	6.76		
Region	Sierra	19.79	6.08	3.949	0.020
	Coast	21.19	6.16		
	Jungle	19.49	6.31		
Area of study	Education and humanities	20.11	6.17	0.541	0.582
	Health sciences	19.66	5.13		
	Engineering	19.44	6.30		
Academic year	First	20.43	6.32	1.029	0.391
	Second	19.76	6.11		
	Third	19.68	5.69		
	Fourth	19.92	6.41		
	Fifth	20.81	6.34		

### 3.3. Somatic Symptoms as a Response to Fear of COVID‐19

Participants in this study reported a moderate level of perceived somatic symptoms in response to fear of COVID‐19 (mean = 7.14, SD = 2.799). Comparison of the study variables using an ANOVA test revealed no statistically significant differences in the manifestation of somatic symptoms, indicating a similarity in the perception of these symptoms between the two groups (Table 3).

**Table 3:** Comparison of perceived somatic symptoms in response to fear of COVID‐19 for each variable.

Variable	Group	Mean	SD	*F* or *t*	*p*
Sex	Female	7.18	2.79	0.763	0.445
	Male	7.00	2.81		
Age	Under 18	6.61	1.94	0.224	0.880
	Between 18 and 25	7.15	2.77		
	Between 26 and 35	7.12	2.98		
	Over 35	7.14	3.15		
Region	Sierra	7.14	2.76	0.361	0.697
	Coast	7.23	2.90		
	Jungle	6.92	2.82		
Area of study	Education and humanities	7.19	2.81	1.010	0.365
	Health sciences	6.71	2.35		
	Engineering	6.87	2.84		
Academic year	First	7.15	2.95	1.713	0.145
	Second	6.96	2.62		
	Third	6.94	2.68		
	Fourth	7.19	2.94		
	Fifth	7.64	2.85		

### 3.4. Psychological Symptoms Manifested as a Response to Fear of COVID‐19

Study participants reported a mean level of perceived psychological symptoms in response to fear of COVID‐19 (mean = 12.89, SD = 3.92). A comparison between the sexes revealed statistically significant differences in the perception of these symptoms between men and women (Table 4). Similarly, the one‐way ANOVA for the geographic region variable indicated differences in the perception of psychological symptoms. Furthermore, post hoc analysis with Tukey′s test (HSD) revealed that students from the Coast perceived a higher level of psychological symptoms of fear compared to students from the Sierra and the Jungle, with a significance of *p* < 0.001 and *p* = 0.021, respectively.

**Table 4:** Comparison of perceived psychological symptoms in response to fear of COVID‐19 between groups for each variable.

Variable	Group	Mean	SD	*F* or *t*	*p*
Sex	Female	13.20	3.83	4.515	< 0.001
	Male	11.80	4.08		
Age	Under 18	12.56	3.40	0.722	0.539
	Between 18 and 25	12.91	3.85		
	Between 26 and 35	12.61	4.37		
	Over 35	13.90	4.60		
Region	Sierra	12.65	3.86	8.048	< 0.001
	Coast	13.96	3.95		
	Jungle	12.57	4.13		
Area of study	Education and humanities	12.92	3.94	0.321	0.725
	Health sciences	12.95	3.33		
	Engineering	12.56	4.10		
Academic year	First	13.28	4.02	0.680	0.606
	Second	12.81	3.97		
	Third	12.73	3.64		
	Fourth	12.73	4.11		
	Fifth	13.17	4.00		

### 3.5. Variables Predicting Fear of COVID‐19

A multiple linear regression analysis was performed to identify predictors of fear of COVID‐19 among Peruvian university students. The regression model included the following independent variables: sex, age, region, study area, and academic year. The results of the analysis are presented in Table 5, which shows the unstandardized coefficients (B), standard error (SE), standardized coefficients (*β*), *t*‐values, and associated *p* values for each predictor.

**Table 5:** Multiple linear regression analysis for variables predicting fear of COVID‐19.

Predictor	B	SE	*Β*	*t*	*p*
Sex	−1.426	0.510	−0.096	−2.794	0.005
Age	−0.017	0.045	−0.014	−0.375	0.708
Region	1.634	0.576	0.172	2.835	0.005
Area of study	−1.440	0.618	−0.142	−2.331	0.020
Academic year	0.188	0.171	0.039	1.101	0.271

Considering a significance level of *p* < 0.05, sex, region, and study area were identified as significant predictors of fear of COVID‐19. *β* indicate the magnitude and direction of each predictor′s relationship with fear of COVID‐19, controlling for the other variables in the model (Table 5). Specifically, being male is associated with a lower level of fear of COVID‐19. The region and study area also showed a significant relationship with fear. In contrast, age and academic year were not significant predictors of fear of COVID‐19 in this model.

The regression model had a multiple *R* of 0.145 and a coefficient of determination (*R* ^2^) of 0.021. These values indicate that the model explains approximately 2.1% of the variance in the fear of COVID‐19 within the sample studied. Although significant predictors were identified, the low variance explained suggests that other factors not included in this model could have a greater influence on the fear experienced by Peruvian university students during the pandemic.

### 3.6. Correlation Between Study Variables

A Pearson correlation matrix was used to assess the statistical relationship between the study variables (Table 6). Strong and statistically significant positive correlations were found between fear of COVID‐19 and both its somatic symptoms (*r* = 0.870, *p* < 0.001) and associated psychological symptoms (*r* = 0.937, *p* < 0.001). This suggests that the greater the fear of COVID‐19 is associated with a higher perception of these symptoms. Additionally, a weak but significant negative correlation was observed between fear of COVID‐19 and sex (*r* = −0.108, *p* = 0.001), indicating a slight trend in the distribution of fear according to sex. Significant, albeit mostly weak, correlations were also observed between other variables such as sex and study area, sex and academic year, age and study area, age and academic year, region and psychological symptoms, region and study area, region and academic year, and study area and academic year.

**Table 6:** Correlation matrix of the relationship between fear of COVID‐19 and other study variables.

Variable	Correlation Coefficient and Sig. (bilateral)	Fear of COVID‐19	Physical symptoms	Psychological symptoms	Sex	Age	Region	Area of study	Academic year
Fear of COVID‐19	*r*	1.000	0.870∗∗	0.937∗∗	−0.108∗∗	0.006	0.053	−0.040	0.025
	*p*	.	< 0.001	< 0.001	0.001	0.852	0.110	0.232	0.447
Physical symptoms	*r*	0.870∗∗	1.000	0.654∗∗	−0.029	−0.008	−0.008	−0.041	0.053
	*p*	< 0.001	.	< 0.001	0.380	0.811	0.806	0.216	0.108
Psychological symptoms	*r*	0.937∗∗	0.654∗∗	1.000	−0.142∗∗	0.013	0.089∗∗	−0.027	−0.001
	*p*	< 0.001	< 0.001	.	< 0.001	0.705	0.007	0.415	0.973
Sex	*r*	—0.108∗∗	−0.029	−0.142∗∗	1.000	0.094∗∗	0.093∗∗	0.221 ^∗∗^	0.011
	*p*	0.001	0.380	< 0.001	.	0.005	0.005	< 0.001	0.750
Age	*r*	0.006	−0.008	0.013	0.094∗∗	1.000	0.141∗∗	−0.091∗∗	0.140∗∗
	*p*	0.852	0.811	0.705	0.005	.	< 0.001	0.006	< 0.001
Region	*r*	0.053	−0.008	0.089∗∗	0.093∗∗	0.141∗∗	1.000	0.663∗∗	−0.228∗∗
	*p*	0.110	0.806	0.007	0.005	<0.001	.	< 0.001	< 0.001
Area of study	*r*	−0.040	−0.041	−0.027	0.221∗∗	−0.091∗∗	0.663∗∗	1.000	−0.225∗∗
	*p*	0.232	0.216	0.415	< 0.001	0.006	< 0.001	.	< 0.001
Academic year	*r*	0.025	0.053	−0.001	0.011	0.140∗∗	‐0.228∗∗	−0.225∗∗	1.000
	*p*	0.447	0.108	0.973	0.750	< 0.001	< 0.001	< 0.001	

*Note:* ∗∗ indicates that the correlation is significant at the 0.01 level (bilateral).

## 4. Discussion

This study was designed to assess the level of fear associated with COVID‐19 among university students in the Coast, Sierra, and Jungle regions of Peru during the first months of the health crisis. The results revealed a moderate level of fear of COVID‐19 in the sample studied. These findings are consistent with other research in similar contexts that found moderate fear (mean = 18.02) in students in Qatar [10], suggesting that the psychological impact of fear was significant in various universities since the beginning of the pandemic [29]. Among university students, fear was exacerbated by the disruption of routines and isolation, intensifying psychological reactions. These findings agree that fear is a primary stressor in young people [30, 31]. Similarly, Machado et al. [32] identified moderate to severe symptoms of depression, anxiety, and stress in university students at the onset of the pandemic, thereby reinforcing the existing level of fear.

The main scientific novelty and contribution of this study lie in its comparative approach to fear of COVID‐19, utilizing a broad and diverse sample of Peruvian university students segmented by geographical regions (Coast, Sierra, and Jungle). Unlike previous research that focused on university populations in other latitudes or in more homogeneous contexts [33, 34], our work offers a national perspective that reveals the heterogeneity of the psychological response to the health crisis in a country with vast geographical and cultural diversity. The analyses revealed a statistically significant variation in the perception of psychological symptoms of fear depending on the students′ geographic region. Specifically, students from the Coast region reported a higher level of psychological symptoms (mean = 13.96) compared to those from the Sierra (mean = 12.65) and the Jungle (mean = 12.57).

The consistency of our findings is supported by existing literature documenting the profound impact of the COVID‐19 pandemic and the resulting containment measures implemented worldwide, which have likely increased the frequency and severity of mental health problems around the world [35, 23]. The adoption of safety protocols, such as social distancing, significantly altered the lives of university students, shifting their academic activities to the virtual world and contributing to the development of mental health problems. This can be seen in the research by Vilca et al. [6], who conducted a study in several cities in Peru that showed that university students′ fear of contracting COVID‐19 influenced the manifestation of symptoms of anxiety, depression, and insomnia. In our study, the overall level of fear found suggests that stress and fear were prominent psychological responses among university students at the onset of the pandemic.

In our study, somatic symptoms, which included physiological manifestations such as sweaty palms, sleep disturbances (insomnia), and tachycardia, were found at a moderate level across the entire study population. In contrast to what was observed for psychological symptoms, our results did not show statistically significant differences in the perception of somatic symptoms between the groups defined by the variables evaluated (*p* > 0.05). Insomnia emerged as a significant factor affecting university students, directly linked to fear of COVID‐19 [36]. This trend is supported by Marelli et al. [37], who reported a prevalence of sleep disturbances (approximately 27%) in nursing students. Similarly, a study of medical students [38] found high rates of depression and poor sleep quality attributed to the pandemic, which, together with our results, underscores the presence of somatic symptoms, highlighting insomnia as a recurring manifestation.

The findings revealed that psychological symptoms, referring to direct fear of COVID‐19, such as distress, fear of death, and information anxiety, significantly predominated (mean = 12.89) over somatic symptoms. Geographically, students from the Coast region (mean = 21.19) reported higher levels of fear than those from the Sierra (mean = 19.79) and the Jungle. This difference between regions is consistent with international studies, such as the one conducted among students in Belarus, Iran, and Russia [39], where fear varied according to nationality. These differences may be due to cultural factors in each country; similarly in Peru, cultural differences are observed at the regional level, may have influenced the perception of fear of COVID‐19.

Our findings suggest the presence of significant emotional effects in the early stages of the pandemic, manifested in feelings of loss and disruption to daily life. This observation is supported by studies documenting an impact on mental health associated with feelings of fear and an abrupt sense of change in daily life stemming from social isolation. It is important to note that the experience of university students during this period was not necessarily negative, as it has been observed that stressful situations can give rise to assessments such as a greater sense of responsibility and care, the strengthening of family interdependence, the promotion of self‐care, and the opportunity for personal reflection on life priorities.

The results reveal that female students reported higher levels of fear than male students [40], expressing anxiety, uncertainty, and loss of control in all three regions of Peru. This qualitative description coincides with previous literature [41], reaffirming that gender influenced the perception of fear of COVID‐19 [20, 42]. Although the area of study was not significant in this analysis, evidence from China [43] indicates that first‐year students experienced greater fear than their peers in advanced cycles. This disparity suggests that specific contextual and temporal factors condition the relationship between academic level and the emotional impact of the pandemic. Post hoc analyses using Tukey′s (HSD) test confirmed that students from the Coast reported higher levels of psychological symptoms of fear than students from the Sierra and the Jungle. This difference was observed in both men and women, underscoring the regional influence on the manifestation of psychological symptoms. In contrast, no statistically significant differences were found in levels of fear or psychological symptoms based on age, year of study, or career trajectory among the participants in the sample studied.

Our study demonstrates that the association between psychological and physical symptoms points to a psychosomatic component as a response to fear of COVID‐19, in which emotional distress is also expressed physically; consequently, these symptoms must be addressed holistically in the context of a health crisis.

Finally, our study found no significant differences between age and fear of COVID‐19, unlike other studies conducted in Colombia [44], which did identify variations in fear levels based on age. It should be noted that younger participants may have had an initial perception of lower vulnerability to COVID‐19, based on the belief that they were in good general health and less likely to experience serious complications; this perspective may have led to an initial underestimation of the potential severity of the disease, treating it as a health problem with limited consequences for their age group. However, it is crucial to recognize that this perception may have evolved over time, depending on the progression and increasing severity of the pandemic, as well as emerging information about its effects on different age groups.

### 4.1. Practical Implications of the Research

The practical implications of this research highlight the need for a multifaceted response from university institutions and public health authorities. First, it underscores the crucial role of university communication platforms in providing accurate and timely information during health crises, fostering the creation of support networks, and combating misinformation. These tools should be used proactively to guide the student community and promote a collective and responsible response to future health emergencies. Second, the findings emphasize the urgency of strengthening and implementing preventive mental health interventions targeting both students and the community at large. These interventions should specifically address fear, anxiety, and other psychological symptoms identified in the study, and should be developed and supervised by mental health professionals. Training healthcare personnel in the timely identification and management of these problems is crucial to mitigating the psychological impact of future crises.

Finally, it is recommended that universities take an active role in promoting mental health by implementing institutional policies that encourage healthy habits and lifestyles. This includes developing evidence‐based programs and collaborating with the Ministry of Health and other relevant organizations to facilitate student access to preventive mental health programs and conferences at the national level. These initiatives should be accessible, culturally sensitive, and tailored to the specific needs of the university population, with the aim of strengthening their resilience in the face of future health crises.

### 4.2. Limitations of the Study

Although this study makes a significant contribution, it is not without limitations. First, the use of convenience sampling due to pandemic restrictions may affect the representativeness of the sample and limit the generalizability of the results to the entire Peruvian student population. Furthermore, the sample is notably unbalanced by gender, with a higher proportion of female participants, which may influence the outcomes and further constrain the external validity of our findings. Second, cross‐sectional design prevents the establishment of definitive causal relationships, allowing only for the inference of associations between variables at a specific point in time. Third, this research did not account for other clinical or psychological variables, such as personality traits, pre‐existing mental health conditions, or specific coping strategies, which could significantly influence fear levels toward COVID‐19. Finally, data collection relied on a single self‐report questionnaire. Although the FCV‐19S demonstrated high internal consistency in this study, the use of a single subjective measure may be subject to social desirability or recall bias. Future longitudinal studies should incorporate a broader range of psychological instruments and more balanced sampling methods to better capture the evolution of mental health outcomes.

## 5. Conclusion

Levels of fear regarding COVID‐19 among Peruvian college students were moderate, with significant disparities based on geographic location and sex. Students from the Coast reported significantly higher levels of fear than those from the Sierra and the Jungle, reflecting an uneven distribution of the pandemic′s psychological impact. Furthermore, across all regions, women reported experiencing greater levels of fear, indicating greater emotional vulnerability and a higher risk of developing mental health problems compared to men. In contrast, variables such as age and academic year had no significant influence on the perception of fear during the initial stage of the crisis.

These findings on the experience of fear of COVID‐19 during the initial phase of the pandemic provide a fundamental basis for future research. These results can serve as a benchmark for comparing postpandemic psychological sequelae among the student population and assessing the long‐term impact of the health crisis. Identifying these at‐risk groups allows for the targeted design of psychological support and prevention programs, optimizing the management of mental health on college campuses. Furthermore, these findings provide an empirical baseline for monitoring postpandemic consequences and strengthening resilience strategies in the face of future global health emergencies.

## Author Contributions

Carmen Y. Baltazar‐Meza and María Custodio: conceptualization, investigation, methodology, writing – original draft, writing – review and editing, supervision, and validation; Patricia Roxana Zarela Quispe‐Baltazar Quispe‐Baltazar and Carmen Soledad Mejía‐Murillo: methodology, investigation, data curation, and formal analysis; Flor Del Rosario Díaz‐Díaz: methodology, investigation, and writing – review and editing. Ciro Lazo‐Salcedo: methodology, investigation, and data curation.

## Funding

No funding was received for this manuscript.

## Disclosure

All authors have read and approved the final version of the manuscripts María Custodio has had full access to all the data from the study and assumes full responsibility for the integrity of the data and the accuracy of the analysis.

## Conflicts of Interest

The authors declare no conflicts of interest.

## Data Availability

The data that support the findings of this study are available from the corresponding author upon reasonable request.
